# A pilot study of Kangaroo mother care in early essential newborn care in resource-limited areas of China: the facilitators and barriers to implementation

**DOI:** 10.1186/s12884-023-05720-4

**Published:** 2023-06-17

**Authors:** Wen Wang, Yinghang Wang, Hanxiyue Zhang, Ge Yang, Yun Lin, Chenran Wang, Xiaona Huang, Xiaobo Tian, Angela Y. Xiao, Tao Xu, Kun Tang

**Affiliations:** 1 Vanke School of Public Health, Tsinghua University, Beijing, China; 2 Institute for Hospital Management, Tsinghua University, Guangdong, China; 3 Division of Neonatology and Center for Newborn Care, Guangzhou Women and Children’s Medical Center, Guangzhou, China; 4 National Center for Women and Children’s Health, Chinese Center for Disease Control and Prevention, Beijing, China; 5 United Nations Children’s Fund Office for China, Beijing, China

**Keywords:** Early essential newborn care, Kangaroo mother care, Qualitative study, China

## Abstract

**Background:**

Implementation of Kangaroo Mother Care (KMC) in resource-limited areas of China may face unique barriers, such as a lack of resources, geographic location and more traditional culture among others. This qualitative study analyses the facilitators and barriers to implementing KMC in county-level health facilities in resource-limited areas of China for the promotion of KMC on a larger scale.

**Methods:**

Participants from 4 of the 18 pilot counties where early essential newborn care was implemented through the Safe Neonatal Project and 4 control counties not enrolled in Safe Neonatal Project were selected using purposive sampling. A total of 155 participants were interviewed, including stakeholders of the Safe Neonatal Project such as national maternal health experts, relevant government officials and medical staff. Thematic analysis was used to process and analyse the interview content in order to summarise the facilitators and barriers to implementing KMC.

**Results:**

KMC was accepted in the pilot areas but still faced certain challenges due to institutional regulation, resource provision and the perceptions of health staff, postpartum mothers and their families as well as COVID-19 prevention and control regulations. The facilitators identified were government officials and medical staff acceptance and the incorporation of KMC into routine clinical care. The barriers identified were a lack of dedicated funding and other resources, the present scope of health insurance and KMC cost-sharing mechanism, providers’ knowledge and practical abilities, parental awareness, postpartum discomfort, fathers’ inadequate involvement, and the impact from COVID-19.

**Conclusion:**

The Safe Neonatal Project pilot experience indicated the feasibility of implementing KMC in more areas of China. Optimising institutional regulations, providing necessary supporting resources and enhancing education and training may help to refine the implementation and scale-up of KMC practice in China.

**Supplementary Information:**

The online version contains supplementary material available at 10.1186/s12884-023-05720-4.

## Background

Over the past 70 years, China has reduced the infant mortality rate from around 200 cases per 1000 births in 1949 to 5.6 cases per 1000 births in 2019 [1], but preterm birth incidence in China has been on the rise, from 5.36% in 1990–1994 to 7.04% in 2016 [2]. Between 2009 and 2018, the proportion of preterm births among newborn deaths has increased from around 42.6–49.8% in China [3]. In addition, immaturity, asphyxia, and congenital anomalies accounted for approximately 80% of preterm newborn deaths during 2009 to 2018 [3]. Although preterm birth complications are still the leading cause of death among newborn in China [4], care for preterm babies remains insufficiently addressed [5]. Kangaroo Mother Care (KMC), including early, continuous, and prolonged skin-to-skin contact between the newborn and mother, exclusive breastfeeding, early discharge from the health facility and close follow-up at home, has been shown to reduce preterm birth complication morbidity, lower preterm or low-birth-weight infant mortality [6, 7], promote breastfeeding [8], enhance the mother-infant relationship and positively impact the infant’s physical and psychological development [9, 10]. For example, a systematic review found that KMC reduced low-birth-weight infants’ mortality rates by 23% compared to conventional care, and increased the likelihood of exclusive breastfeeding by 50% [7]. In 2014, the Department of Maternal and Child Health of the National Health Commission of China established the Premature Birth and Preterm Infants Intervention, recruiting 10 hospitals to pilot KMC implementation [11]. From 2014 to 2018, the Premature Birth and Preterm Infants Intervention actively raised awareness and promoted KMC amongst its network of 50 hospitals [11, 12], and a 12-month prospective multicenter study was conducted in 8 of these hospitals [13]. In 2017, the “Guidelines for Health Care Services for Premature Infants” was released, which further promoted the scale-up of KMC in China [14]. Despite these efforts, KMC is still not widely practiced in China. It was only being implemented in some selected hospitals where it was introduced through some specific pilot project, and has not yet been incorporated into routine clinical practice in China.

China have taken actions and have marked improvements in children’s health over the past few decades [15], but wide regional disparities in the burden of newborn deaths still persist in China. In 2020, the neonatal mortality rates were 4.8‰ in western China and 1.8‰ in eastern regions [16]. The western regions also has the highest preterm birth rate [2]. The significant resource gap between eastern and western China further exacerbates disparities in neonatal health. Since KMC is relatively cost-effective and can be delivered in low-resource settings, introducing KMC to resource-limited, western China may be an effective strategy to improve neonatal outcomes. A study conducted in Bogotá shows that the incremental cost-utility ratios of prolonged in-hospital care in incubators at one year of corrected age was $ 1,546 per extra quality-adjusted life year compared to the KMC method [17]. In some resource-limited areas of China, usage of certain neonatal intensive care unit (NICU) equipment (e.g., incubators) is minimal due to the high upfront costs and maintenance demands. KMC, in contrast, does not require such equipment; for example, skin-to-skin contact can be achieved by wrapping the infant in the mother’s clothes. Thus, KMC may also serve as a sustainable healthcare solution for low-resource settings.

In an effort to improve neonatal outcomes and minimize regional disparities, the National Health Commission of China and the United Nations Children’s Fund (UNICEF) jointly launched the Safe Neonatal Project from September 2017 to December 2020 with the aim to deliver early essential newborn care (EENC) to all hospitals within the pilot areas across 18 counties in four western Chinese provinces. KMC implementation was one of the optional project interventions in the Safe Neonatal Project.

Although there are many studies focusing on the facilitators and barriers to KMC implementation worldwide, we assumed that western China has certain country and regional specificities compared to the eastern and central regions— namely its environmental, economic and social specificities— which may create unique barriers to KMC practice. For example, compared to the eastern coastal region, certain areas of western China have limited economic resources and a lack of medical reserves [18]; some mountainous areas have long winters and a harsher climate; and some autonomous regions have populations made up of mainly ethnic minority groups with unique cultural customs and identities which may increase health inequality in maternal and child health [19, 20]. Thus, we conducted this qualitative study to examine the barriers and facilitators to KMC implementation in low-resource settings and propose suggestions to improve KMC scale-up in China.

## Method

### Study design and site

The present study is part of an evaluation project, which aims to document implementation of the Safe Neonatal Project by assessing the acceptability, sustainability and scale-up of implementing EENC in low-resource areas of China as well as its barriers and facilitators. Throughout the Safe Neonatal Project, pilot areas followed the practical guide issued by World Health Organization (WHO) [21] while also gaining their own insights and experience with KMC implementation.

From December 2020 to April 2021, we conducted qualitative research to identify the barriers and facilitators to KMC implementation. This study randomly selected four pilot counties from Qinghai Province, Sichuan Province, Guizhou Province and Ningxia Hui Autonomous Region, among the 18 counties that conducted the Safe Neonatal Project. Within Qinghai, Sichuan, Guizhou and Ningxia Hui Autonomous Region, four control counties homogeneous in socioeconomic development, demography and maternal and child health care levels to each of the pilot counties and which were not enrolled in Safe Neonatal Project were selected. One to two county-level hospitals that both provide midwifery services and have an annual record of around and over 1000 live births were chosen as sample health facilities in each pilot and control county (the characteristics of the health facilities are shown in Appendix Table A). Health facilities in the pilot areas implemented EENC, while the control areas implemented conventional neonatal care. (Table 1)

**Table 1:** Research sites

Site	Code
Pilot- Qinghai Province	PQ
Pilot- Sichuan Province	PS
Pilot- Guizhou Province	PG
Pilot- Ningxia Hui Autonomous Region	PN
Control- Qinghai Province	CQ
Control- Sichuan Province	CS
Control- Guizhou Province	CG
Control- Ningxia Hui Autonomous Region	CN

### Participants and sampling

Purposive sampling was performed to recruit participants, including policy-makers, national EENC expert representatives, obstetricians, pediatricians and nurses. In order to ensure full representation of the different stakeholders involved in KMC implementation, county mayors in charge of health, county mayors in charge of finance, hospital medical directors, obstetricians, pediatricians, and nurses from each county and each hospital were selected purposively. The sample size was determined as per data saturation [22, 23]. We compared the data collected in the later interviews with the data from earlier interviews to check if new information was emerging or if the same information was repeating. If no new information was discovered, it suggested that saturation had been achieved and our sample size was sufficient.

### Data collection

This study collected data using semi-structured interview outlines, and organised seven in-depth interviews (IDIs) for national EENC expert and 22 focus group discussions (FGDs) for policy-makers and medical staff. A total of 155 interviewees were included: 7 national experts, 54 policy-makers in charge of the county-level government and county health and finance departments (26 in pilot areas, and 28 in control areas) and 94 medical staff (45 in pilot areas, and 49 in control areas) (Appendix Table B shows the sample size in each county). Information sheets detailing the study purpose, risks and anonymity and confidentiality assurances were provided before the start of the interview to participants. Consent forms were read and signed by all participants who agreed to partake in the study. The interview outlines were designed by the research team before the study and revised dynamically throughout the multiple rounds of interviews. The main interview content included (1) whether KMC was conducted with the implementation of EENC or planned to be implemented later; (2) the practice and experience of KMC implementation in the pilot areas or the willingness to implement KMC in the control areas; and (3) the potential barriers and facilitators in KMC implementation.

IDIs and FGDs were conducted by trained facilitators and note takers in Chinese. To ensure stakeholders could provide their subjective assessments of the implementation of KMC based on their experiences, all interviews were conducted in private and quiet rooms in hospitals of the pilot and control counties. Each of the IDIs lasted 50 to 60 min; FGDs lasted 90 to 100 min. Interviews were audio-recorded with the consent of the interviewees. Recordings were transcribed verbatim by the researchers; private information was de-identified to form anonymous interview transcripts. The recordings were reviewed and the transcribed data re-read several times to ensure the accuracy of the transcription. All recordings and interview transcripts were for the researchers’ access only. Ethical approval for this study was obtained from the Institutional Review Board of the National Center for Women and Children Health, Chinese Center for Disease Control and Prevention (FY2019-09).

### Data analysis

This study used a thematic analysis method for data analysis [24], following the inductive approach. Two independent researchers used three levels of coding simultaneously to gradually extract basic semantic codes from the IDI and FGD transcripts. Researchers first read through the data separately, noting early thoughts; then we used line-by-line coding to examine each part of the data. After synthesising basic semantic codes into organising codes (e.g. “acceptable”, “guidelines”, “resource allocation”, “perceptions”), researchers cross-combined the codes to verify possible contradictions and extract themes. To ensure the trustworthiness of the study findings, we adopted an 80% minimum agreement between coders. Finally, six themes emerged from the organising codes: acceptability, availability, pre-preparing, affordability, individuals, COVID-19 (Appendix Figure A shows the thematic map generalised from the data). QSR Nvivo 12.0 was used for data generalisation and numerical coding.

### Techniques to enhance trustworthiness

Triangulation was used to strengthen the credibility and validity of our research. Both data triangulation and investigator triangulation were used. In this study, data was collected from stakeholders from different districts and backgrounds for mutual authentication and by several trained researchers from different backgrounds. To verify data credibility, we used participant validation of the findings. We reported our research findings to some of the national experts who participated in the IDIs and examined their review of and feedback on the findings to verify the accuracy and relevance.

After each data collection session, we immediately debriefed with the data collectors to gain a comprehensive understanding of the content of the data collected and dynamically revised interview outlines to strengthen the quality and trustworthiness of data in real time. Adjustments were made in response to challenges and unforeseen events.

The codes and themes were reviewed periodically throughout data collection and analysis to ensure that the findings were based on the participants’ responses rather than potential researcher biases. We also had the audit trail detailing each process of data collection, data analysis, and data interpretation during our research.

## Results

### Basic characteristics of the study participants

There were 155 participants interviewed in this study who represent the different roles of stakeholders in KMC implementation, including representatives of UNICEF and the Chinese national health authority, county mayors, hospital medical directors, nurses, obstetricians, paediatricians, etc. The participant characteristics are shown in Table 2.

**Table 2:** Participant characteristics

Stakeholder type		FGD or IDI	Number of participants	Sex	Years of EENC enrollment/experience
EENC national experts	UNICEF representative	IDI	1	Male	4 years
	National Health Commission of China representative	IDIs	2	50% female	4 years
	National EENC specialist	IDIs	4	All female	4 years
Policy-makers	County mayor	FGDs	5	All male	N/A
	Deputy county mayor in charge of health	FGDs	8	63% male	N/A
	Others (office of health, finance, development and reform, et al.)	FGDs	17	71% male	N/A
	Hospital medical director	FGDs	24	67% male	2–3 years
Medical staff	Nurse	FGDs	29	93% female	2–3 years
	Paediatrician	FGDs	20	60% female	2–3 years
	Obstetrician	FGDs	33	82% female	2–3 years
	Others (e.g. hospital-acquired infection control department)	FGDs	12	75% female	2–3 years

### Summary of facilitators and barriers to KMC implementation

One set of data related to the facilitators of KMC implementation, and the other set related to the barriers of KMC implementation. Given the disparate directions of the two sets, the findings are organized accordingly. The thematic analysis process resulted in the emergence of 20 organising codes and six themes, which can be found in Appendix Figure A. Appendix Figure B provides the frequency of appearance for each theme across all 29 interviews (7 IDIs and 22 FGDs). Among them, the majority view captured was on the theme of “acceptability” and “pre-preparing”. The barriers and facilitators to KMC implementation described by participants included the acceptability, availability, affordability of KMC as well as certain characteristics of individuals and the COVID-19 lockdown period, which are detailed in Table 3 and discussed below.

**Table 3:** Identified barriers and facilitators to KMC implementation

Themes	Codes						
	Facilitators		Barriers				
Acceptability of KMC service	accepted by government officials	viewed positively by medical staff					
Availability of KMC service	successfully implemented in some of the pilot counties	incorporated KMC into clinical process					
Pre-preparing for KMC implementation	learned KMC in other trainings	Attempt to localized the KMC practice based on local medical conditions	lack of Chinese- localised KMC guidelines and policies	inadequate infrastructure	inadequate equipment	lack of consumables	medical staff need more training in KMC practice
Affordability of KMC service			lack of dedicated funding	not included in the scope of health insurance	no cost-sharing mechanism for the resources consumed by KMC implementation		
Individual level factors			some medical staff did not have adequate knowledge	low awareness of KMC among parents	postpartum discomfort	lack of fathers’ involvement	
COVID-19 lockdown period			restricted access to neonatal intensive care units and maternity ward during COVID-19				

### Facilitators to KMC implementation

#### Acceptability of KMC service

Participation in the Safe Neonatal Project and provision of relative training were two main facilitators that increased acceptance of KMC. Since the launch of the Safe Neonatal Project, KMC has been accepted by local medical staff and government officials and successfully implemented in some of the pilot counties. Two of the four pilot counties have implemented KMC for some preterm infants. A site in Sichuan saw an increase in KMC acceptance among medical staff following successful in-hospital cases, with an implementation rate of 60–70%.

#### Pre-preparing for KMC implementation

Some participants noted that the Safe Neonatal Project greatly facilitated KMC implementation, with increased acceptance of KMC reported after receiving the EENC training. Medical staff in the control counties also had a positive view of KMC. Some medical staff reported that although their institutions were not included in the EENC pilot, they had learned KMC in other trainings, which increased their acceptance of KMC.

*“After we received the EENC training, then we [were] more accepting of KMC for premature babies.“ (PS10 - medical staff)*.

*“KMC was advocated for and discussed in our hospital a long time ago, but we just couldn’t do it. However, since participating in the Safe Neonatal Project, we are now reaching 60–70% [implementation], which is very good rate.“ (PS13 - medical staff)*.

#### Availability of KMC service

People localized KMC practice according to their local medical conditions, which helped to enhance the potential availability of KMC in resource-limited areas of China. One pilot county designed its own neonatal straps for KMC and plans to conduct a trial run in the future. In one control county, a local health facility who had already incorporated KMC into its clinical process provided a KMC designated area in the delivery room for two hours of post-delivery to facilitate KMC practice.

*“Our obstetrics department designed a consumable, which was made by knotting the hem of a pair of large pyjamas. This can help the mother stay warm when conducting KMC. The pyjama shirt has buttons to fasten it up and wrap the baby in it, and the elastic band is adjustable, so the baby won’t fall out. We have already designed this, but haven’t tried it yet.“ (PN8 - medical staff)*.

*“We will call the newborn’s family in advance to set a time and check their convenience. The mother will change into suitable clothes at home, with pyjamas on the inside and a soft robe outside. When the mother goes to the hospital, we will prepare a recliner, which can be used sitting or lying down…we also have special rooms and other supporting facilities.“(CS6 - medical staff)*.

*“Now we keep the mother and baby in the same room, and then conduct KMC…our delivery room now has a designated area for KMC. After delivery, mothers and their babies stay there for two hours.“ (CS12 - medical staff)*.

### Barriers of KMC implementation

#### Pre-preparing for KMC implementation - lack of localised guidelines and policies

The lack of Chinese-localised KMC guidelines and policies was an important factor limiting implementation of KMC in resource-limited areas of China. The main reference and authoritative guidelines previously used in China were the English-version guidelines issued by WHO in 2013 [21], which are less accessible for medical staff based in primary care health facilities. During the Safe Neonatal Project implementation period, medical staff also relied on the Chinese EENC expert consensus [25] which only briefly mentions KMC, to guide the training and practice. However, since neither health care administration offices nor professional associations in China issued specialised clinical practice guidelines for KMC during the Safe Neonatal Project implementation period, participants indicated that there was a need for standardised Chinese-localised implementation practices that can help guide primary care medical staff and advise on quality control.

*“When we went to the grassroots [primary care health facilities], we found that [primary care medical staff] feel that just the EENC expert consensus is not enough. They’re hoping for implementation guidelines to be released and believe it would be better if KMC can be incorporated into medical textbooks. After this, [primary care medical staff] will have more confidence to implement.“ (National experts 2)*.

#### Pre-preparing for KMC implementation - inadequate resources allocation in resource-limited areas of China

A lack of resources, including infrastructure, equipment, consumables, and funding, was also a significant factor limiting implementation of KMC.

Inadequate infrastructure and low-quality equipment in some of the health facilities further restricted the implementation of KMC. For example, some study areas are located in the northwest of China, where winters are long and harsh. Only some health facilities in these areas were equipped with good heating systems, so during the winter, room temperatures sometimes dropped below the minimum temperature necessary for conducting KMC. In addition, some study areas had a limited number of delivery rooms. Hospitals may have struggled to guarantee a safe and private space for patients, which may discourage mothers from accepting KMC.

*“Our delivery room is a bit rudimentary, and with the cold climate here, we won’t carry out KMC if the temperature does not reach the acceptable range.“ (CG6 - medical staff)*.

*“In terms of KMC, one of the basic requirements is a private environment. It requires mothers to be in partial nudity, so ensuring their privacy is essential. We must have a separate space for the mother to conduct KMC.“ (PS2 - health administration officer)*.

A lack of supplies was reported in this study by some healthcare providers working in KMC units. In terms of equipment and consumables, previous studies have suggested that standardised equipment (e.g., recliners) and consumables (e.g., neonatal straps) should be provided in order to ensure both the comfort of mothers and infants as well as the safety of conducting KMC [26, 27]. There are few studies examining the status of standardised equipment and consumables for KMC in China. Standardised neonatal straps used to secure newborns to their mothers were specifically mentioned several times by medical staff in the pilot areas.

*“I think the KMC consumables need to reach a higher level of quality. Although we developed our own (straps), the quality has not been guaranteed yet. We are afraid to try it directly on our patients.“ (PQ4 - medical staff)*.

#### Affordability of KMC service

At present, KMC is not a billable item under government regulations and thus, is not included in the scope of health insurance. Health facilities have limited funding resources to support the costs of KMC. Although the implementation of KMC requires relatively few consumables or equipment, it requires the assistance and support of doctors or nurses, which increases workloads and generates additional labour costs. Currently, there is no cost-sharing mechanism for the resources consumed by KMC implementation. During the pilot period, the costs of consumables or equipment were mostly borne by the hospitals themselves, and no compensation was provided to medical staff for their additional labour. In addition, while medical staff indicated that limited delivery room availability restricted KMC implementation, they also said that hospital renovations were not possible due to insufficient funding.

*“Our government does not plan to include the cost of KMC in basic health insurance, so we cannot charge [patients] for it through health insurance. Although, for the patient, this service is provided free-of-charge, but the actual costs of providing KMC are consuming our hospital’s limited funding.“ (PS5 - health administration officer)*.

#### Individual level factors

While some medical staff already had prior knowledge of KMC and believed it to be a simple and feasible way to care for preterm infants, others still doubted the feasibility of introducing KMC practice to their own hospitals. In particular, some expressed uncertainty about the indications for implementing KMC in preterm infants. Provider training to increase knowledge and competence was mentioned as a strategy to help eliminate concerns and improve attitudes towards KMC.

*“We did not conduct KMC. Mothers were unwilling to hold their babies for a long time. We didn’t have too many preterm births. We don’t know the clinical indications of KMC for preterm babies to be able to do this. We generally hold children who are born prematurely in incubators.“ (PN11 - medical staff)*.

*“There are actually some [ideological] obstacles for our medical staff. [Acceptance of KMC] may require a long time and continuous reinforcement.“ (PS3 - medical staff)*.

The perceptions of mothers and their families was also found to influence the implementation of KMC. Mothers and their families in the pilot areas did not know much about KMC, believing that it would be better for preterm infants to stay in incubators, and thus, were unwilling to accept KMC. Some mothers did not accept implementation of KMC due to postpartum discomfort, significant pain and the interference of KMC with their rest.

*“Many of our patients are rural people with relatively low educational attainment. The inculcation of new knowledge and concepts is a long process, and it has only been two and a half years [since the EENC pilot].“ (PS8 - medical staff)*.

*“Some of the mothers had cesarean sections, others were bleeding, and still others were in acute pain and could not hold [the baby] or were nervous about caring for low-birth weight babies with their own bodies.“ (PS14 - medical staff)*.

*“Mothers did not want to hold their babies for extended periods of time.“ (PN3 - medical staff)*.

Furthermore, in some pilot areas, the lack of father’s involvement also hampered KMC implementation. Medical staff said that some fathers were deeply influenced by traditional local customs, believing that the responsibility to care for the child was solely the mothers’, and thus were unwilling to participate in KMC. However, when only mothers participated in KMC, implementation was often less smooth and timely.

*“I think fathers may not be able to do this, because most of them think caring for the child is the mother’s responsibility…” (PQ3 - medical staff)*.

#### COVID-19 lockdown period

During the pandemic, some health facilities strengthened COVID-19 prevention and control measures. They restricted access to neonatal intensive care units and prohibited drop-in visits or required negative nucleic acid test results to make appointments, which interfered with parents’ ability to enter the maternity ward and conduct KMC.

*“Before the pandemic, all the pilot areas had originally intended on implementing KMC. But now, many are no longer participating. Going to the ward requires nucleic acid [testing]…and, in fact, medical facilities must also shoulder a very high risk, because other children are also present in neonatal intensive care units, so [implementing KMC] may increase the potential risk of COVID transmission” (National experts 2)*.

## Discussion

This study is the first study evaluating KMC implementation in resource- limited areas of China since KMC integrated into EENC practice. This study identified barriers and facilitators to KMC implementation in resource-limited areas of China. After carrying out several IDIs and FGDs, several facilitators and barriers emerged: the acceptability and potentially available of KMC; a lack of localised guidelines and policies; inadequate allocation of resources; the need for improved medical staff and patient knowledge and awareness; and the impacts of the COVID-19 pandemic. In order to promote KMC implementation across county-level health facilities in resource-limited areas of China, it is recommended to take these facilitators and barriers into account when designing and adopting implementation strategies to scale up interventions.

KMC is an important intervention that can enhance the health of newborns and promote early childhood development [28]. In this study, we found that KMC has been endorsed by health facilities in the Safe Neonatal Project pilot areas. Most of the medical staff in the pilot areas expressed a willingness to learn and implement KMC. Several pilot areas have implemented KMC for some preterm infants impromptu. In accordance with the results of other studies, this study found that training medical staff is a potential facilitator that can improve the acceptability of KMC. Previous studies have shown that the training received by healthcare providers is crucial for improving their knowledge and ability to support KMC implementation [29–31]. This study found that even if the training is not KMC-specific, the inclusion of KMC content in other neonatal care related training may greatly promote the acceptance of KMC among medical staff. Additionally, this study found that adaptation of KMC to the local context is a potential facilitator. For example, most of the previous countries that have conducted KMC are located near the equator and are not particularly cold even in winter. Thus, there is no need for certain supplies, such as a pyjama shirt to help wrap the baby and warm up the mother. In this study, participants in certain areas localised the KMC practice unprompted, adding pyjamas, neonatal straps, etc. as supplies. These changes facilitated the implementation of KMC in colder resource-limited areas.

The policy environment was found to be a critical factor affecting implementation of KMC. In terms of clinical practice, WHO specified that KMC implementation requires the establishment of clinical guides, with the participation of local professional groups, to ensure standardised implementation of KMC [32]. In late 2022, a Chinese clinical practice guideline for KMC in preterm and low birth weight infants was released [33]. We recommend that government agencies and hospitals support the use of this localised clinical guideline in order to prevent medical safety risks, raise the confidence of medical staff in implementing facilities to carry out KMC, and save time and labour costs for KMC training. In terms of health systems, improving KMC implementation requires the national government and health facilities to amend the existing health policy system. In order to promote the incorporation of KMC into the routine clinical practice for preterm and low birth weight babies, the service cost and health insurance payment system must be optimised. A study found that the major cost driver of KMC is human resources, followed by training and supplies [34]. Considering the inadequacy of the present scope of health insurance and KMC cost-sharing mechanism, we recommend the government adjusting medical insurance coverage to better reflect the labour costs, investing in initial and recurrent KMC training for medical staff and providing assistance to the necessary hospital facility reforms.

Inadequate funding and poor resource allocation in county-level health facilities in western China further limits the implementation of KMC. In many countries, KMC is often not considered a priority for hospitals and limited resources are allocated to it [29, 35]. Studies have shown that funding, equipment and labour are key factors affecting the implementation of KMC [36, 37]. Improved resource allocation and adaptation to local contexts are needed to improve KMC implementation in county-level health facilities in western China. Hospital administrators and government officials should invest more resources towards improving the space and overall environment of delivery rooms to meet the privacy and comfort needs of mothers or other family members. Previous studies in developing countries such as Uganda [38], Ethiopia [39] and Ghana [40] have shown that government or hospital investments aimed at improving the privacy and comfort of the delivery environment have improved KMC implementation. With regards to labour resources, uneven and inadequate regional allocation of medical staff may negatively affect KMC implementation [41, 42]. As for equipment, ensuring the supply of standardised, safe and comfortable equipment and consumables can enhance parental compliance with KMC implementation [43]. It is recommended that the government or health management institutions assist county-level health facilities in procuring safe and low-cost straps by issuing unified standards, specifying the materials and specifications of KMC straps, and providing dedicated funds for hospitals to purchase equipment or helping them seek the necessary funding.

In this study, we found that medical staff in some pilot areas did not have sufficient hands-on experience with KMC and lacked the confidence to carry out KMC. Improving training and increasing the participation of relevant stakeholders, such as medical staff, hospital managers, mothers and their families, may help promote KMC implementation [12, 44]. Experience from other countries has shown that strategies to help sustainably improve medical staff’s confidence in implementing KMC and avoid overuse of neonatal intensive care units include enhancing training and supervision, improving communication between doctors and nurses and increasing contact between hospital management and the clinical team [45–47].

In addition, the promotion of KMC also requires the support and understanding of mothers and their families. Previous studies have shown that there are similar physiological and biochemical responses in preterm neonates no matter when KMC conducted by mother or father [48, 49]. Besides, joint parental participation in KMC can help reduce the mental stress and fatigue of mothers [33]. Considering the traditional local customs in some regions and the discomfort feeling of postpartum mother’s reported by healthcare professionals, we recommend increasing the role of the father in KMC implementation to enhance the father’s parenting confidence and benefit both parties. Involving both parents in KMC is also promoted in the Chinese KMC guideline [33]. Moreover, studies have shown that educational programmes are paramount to increasing parental knowledge, and thus improving uptake of KMC [33, 50]. To help counter widespread misconceptions in some regions, it is recommended to strengthen KMC education for parents also through social media or other channels.

There were several limitations in this study. First, we acknowledge the possibility of social desirability bias from medical staff when participating in the interviews. To minimise this bias, we used non-hospital staff as researchers to create a relatively free environment for people to speak their minds. Second, because some resource-limited areas were selected as EENC pilot sites, this study may have limited generalisability throughout China, especially for the affluent eastern coastal regions. Third, since KMC was an optional rather than mandatory intervention of the Safe Neonatal Project, the research sites may not have devoted the same number of resources and training to implementing KMC as they did to mandatory EENC interventions, which may undermine the acceptance and implementation rate of KMC in the pilot areas. Lastly, regarding participant validation of the findings, we only included the views of some national experts. In future studies, we hope to include the views of multiple stakeholders to further enhance the credibility of the data.

## Conclusion

KMC is a cost-effective intervention that can be widely promoted in resource-limited areas of China. It has been endorsed by local governments and health facilities in the Safe Neonatal Project pilot areas. Yet, KMC implementation still faces challenges in terms of the policy environment, human and financial resources and doctor-patient perceptions. By promoting Chinese-localised KMC clinical guidelines, optimising service processes, investing in health resources and strengthening education and training for medical staff, pregnant women and their families, a more favourable environment for the implementation and scale-up of KMC can be cultivated.

## Electronic supplementary material

Below is the link to the electronic supplementary material.

**Additional file 1:** Appendix Table A

**Additional file 2:** Appendix Table B

**Additional file 3:** Appendix Figure A

**Additional file 4:** Appendix Figure B

## Data Availability

The datasets supporting the conclusions of this article are not publicly available but are available from the corresponding author on reasonable request.

## Abbreviations

EENC: Early Essential Newborn Care
FGD: Focus Group Discussion
IDI: In-Depth Interview
KMC: Kangaroo Mother Care
NICU: Neonatal Intensive Care Unit
UNICEF: United Nations Children’s Fund
WHO: World Health Organization
