# Supplementary material for: A pilot study of Kangaroo mother care in early essential newborn care in resource-limited areas of China: the facilitators and barriers to implementation
Source: BMC Pregnancy Childbirth. 2023 Jun 17;23:451. doi: 10.1186/s12884-023-05720-4 (PMC10276515; doi:10.1186/s12884-023-05720-4)
Supplement: Supplementary file 1 — Additional file 1: Appendix Table A [file 12884_2023_5720_MOESM1_ESM.docx]

Appendix

Table A The characteristics of health facilities conducting EENC in selected 4 pilot and 4 control counties

| Number | Site code | Classification (grade and level) | Hospital bed numbers | Medical staff numbers | Doctors number | Nurses number | Annual number of deliveries | Annual number of live births |
| --- | --- | --- | --- | --- | --- | --- | --- | --- |
| 1 | PQ | Grade III Level B | 68 | 32 | 13 | 19 | 920 | 920 |
| 2 |  | N/A | 8 | 11 | 5 | 6 | 16 | 16 |
| 3 | CQ | Grade II Level A | 257 | 382 | 72 | 145 | 1183 | 1182 |
| 4 |  | Grade III Level B | 422 | 585 | 263 | 250 | 1211 | 1211 |
| 5 | PS | Grade II Level A | 91 | 253 | 79 | 109 | 1926 | 1926 |
| 6 |  | Grade III Level B | 850 | 762 | 204 | 358 | 1141 | 1133 |
| 7 | CS | Grade III Level B | 950 | 1255 | 342 | 583 | 1679 | 1636 |
| 8 |  | Grade II Level A | 105 | 271 | 79 | 114 | 1826 | 1832 |
| 9 | PG | Grade II | 100 | 88 | 14 | 40 | 144 | 144 |
| 10 |  | Grade II Level A | 385 | 557 | 156 | 260 | 1906 | 1904 |
| 11 | CG | Grade II Level A | 410 | 643 | 259 | 280 | 2020 | 2030 |
| 12 |  | N/A | 60 | 67 | 21 | 25 | 187 | 186 |
| 13 | PN | Grade II | 25 | 54 | 26 | 13 | 287 | 289 |
| 14 |  | Grade II Level A | 398 | 469 | 111 | 233 | 575 | 577 |
| 15 | CN | Grade II Level A | 25 | 459 | 148 | 207 | 736 | 743 |
| 16 |  | Grade II Level B | 118 | 155 | 27 | 74 | 676 | 676 |
