# Supplementary material for: A pilot study of Kangaroo mother care in early essential newborn care in resource-limited areas of China: the facilitators and barriers to implementation
Source: BMC Pregnancy Childbirth. 2023 Jun 17;23:451. doi: 10.1186/s12884-023-05720-4 (PMC10276515; doi:10.1186/s12884-023-05720-4)
Supplement: Supplementary file 2 — Additional file 2: Appendix Table B [file 12884_2023_5720_MOESM2_ESM.docx]

Appendix

Table B Sample size of interviewees

| Stakeholders | Total | | PQ | CQ | PS | CS | PG | CG | PN | CN |
| --- | --- | --- | --- | --- | --- | --- | --- | --- | --- | --- |
|  | Pilot | Control |  |  |  |  |  |  |  |  |
| National experts | 7 | |  |  |  |  |  |  |  |  |
| Policy-makers | 26 | 28 | 5 | 8 | 9 | 7 | 7 | 7 | 5 | 6 |
| Medical staff | 45 | 49 | 6 | 12 | 18 | 16 | 9 | 6 | 12 | 15 |
