# Supplementary figures and images for: A pilot study of Kangaroo mother care in early essential newborn care in resource-limited areas of China: the facilitators and barriers to implementation
Source: BMC Pregnancy Childbirth. 2023 Jun 17;23:451. doi: 10.1186/s12884-023-05720-4 (PMC10276515; doi:10.1186/s12884-023-05720-4)

Appendix

Figure A Thematic framework map


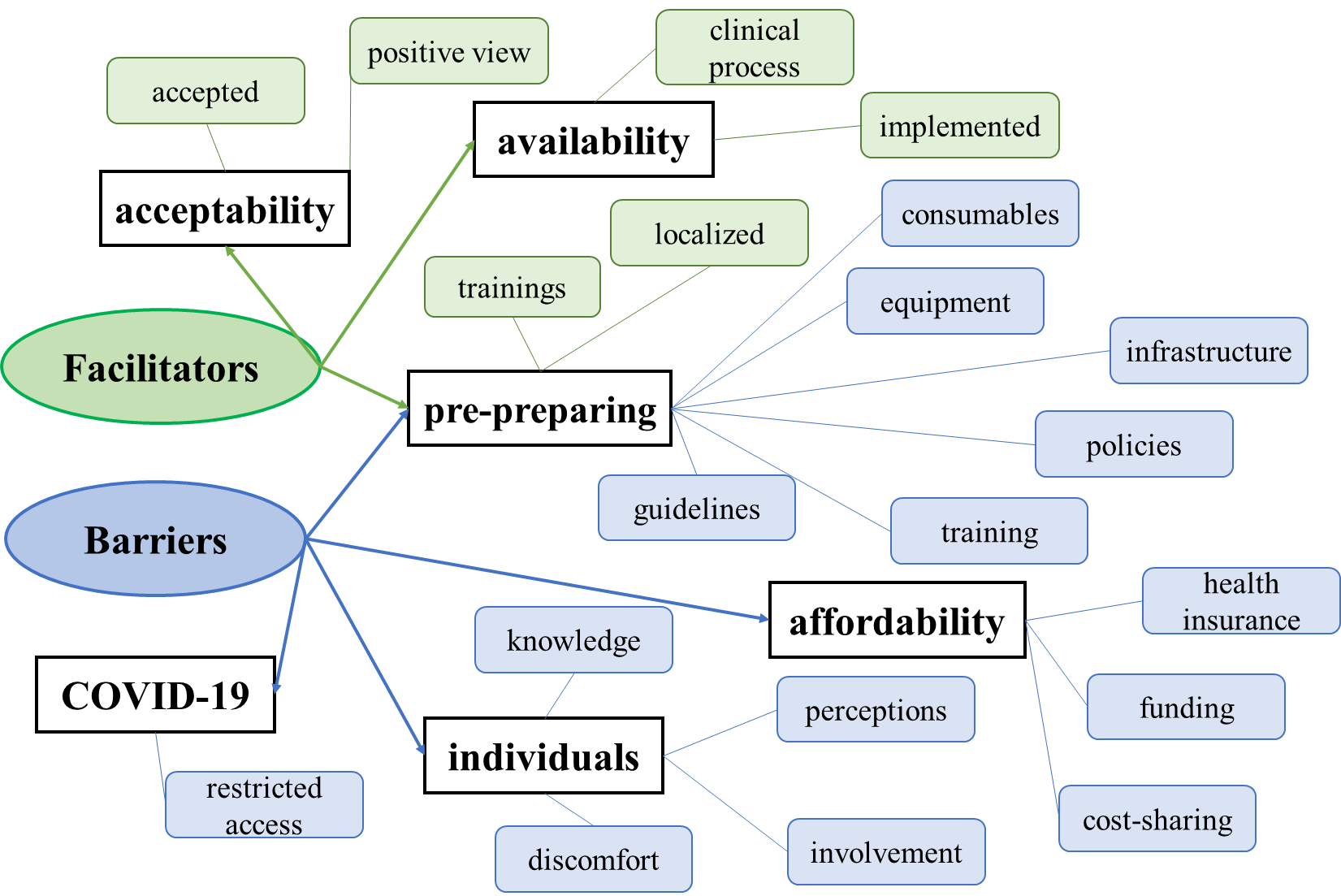

Supplement: Supplementary file 3 — Additional file 3: Appendix Figure A [file 12884_2023_5720_MOESM3_ESM.docx]

Appendix

Figure B The frequency of each theme appeared among 29 interviews (7 IDIs and 22 FGDs)


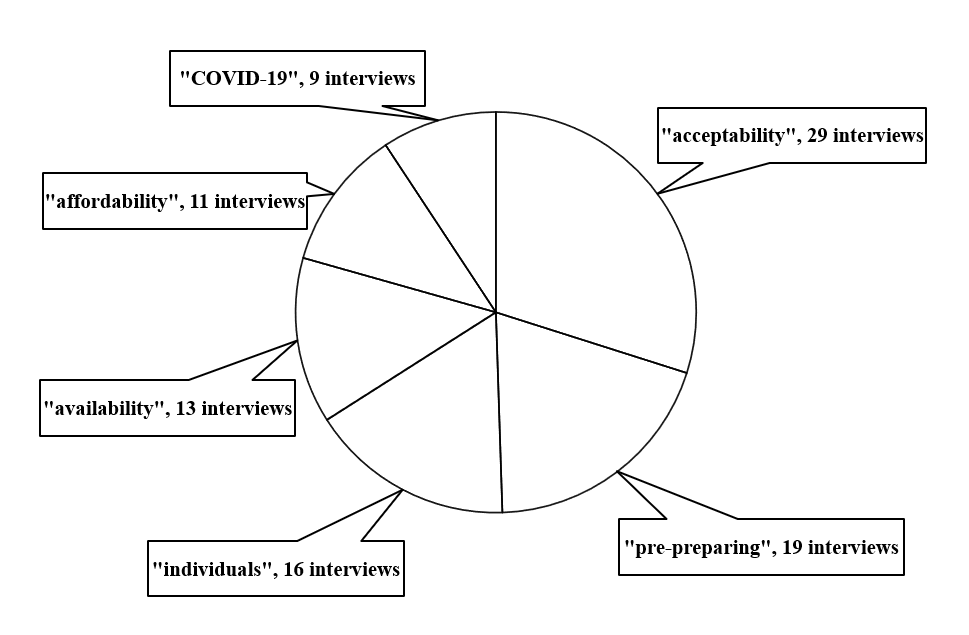

Supplement: Supplementary file 4 — Additional file 4: Appendix Figure B [file 12884_2023_5720_MOESM4_ESM.docx]
